# A systematic review of the impact of therapeutic education programs on the quality of life of people with Multiple Sclerosis

**DOI:** 10.34172/hpp.42619

**Published:** 2024-07-29

**Authors:** Ilham Raji, Ibtissam El Harch, Mohammed El Amine Ragala, Mohamed Berraho, Fedwa Nejjar, Mohammed Faouzi Belahsen

**Affiliations:** ^1^Laboratory of Epidemiology and Health Sciences Research, Faculty of Medicine, Pharmacy and Dentistry, Sidi Mohammed Ben Abdallah University, Fez, Morocco; ^2^Department of Neurology, Hassan II University Hospital Center, Fez, Morocco; ^3^Laboratory of Epidemiology, Clinical Research and Community Health, Faculty of Medicine, Pharmacy and Dentistry, Sidi Mohammed Ben Abdallah University, Fez, Morocco; ^4^Laboratory of Natural Substances, Pharmacology, Environment, Modeling, Health and Quality of Life, Faculty of Sciences Dhar El Mahraz, Sidi Mohamed Ben Abdellah University, Fez, Morocco; ^5^Department of Biology and Geology, Teachers Training College (Ecole Normale Superieure), Sidi Mohamed Ben Abdellah University, Fez, Morocco

**Keywords:** Multiple sclerosis, Educational programs, Quality of life, Therapeutic education

## Abstract

**Background:**

Faced with a deemed mediocre quality of life (QoL) in people with multiple sclerosis (pwMS), the effectiveness of therapeutic education (TPE) programs is called into question. This systematic review is conducted to examine the impact of the TPE programs on the QoL of pwMS.

**Methods:**

A search was performed in three databases (PubMed, Web of Science and Scopus) to identify relevant studies published between 2007 and 2022. The review followed the PRISMA guidelines. Two reviewers independently extracted data on the study and program characteristics. These data were presented in tables for detailed synthesis and descriptive analyses. The selected studies underwent assessment using recommended evaluation tools.

**Results:**

Of the 21 studies included in the review, 13 found a significant improvement in QoL, which was maintained during follow-up testing in 42% of the studies. TPE programs that focused on patients’ individual needs and aimed to develop their skills in a personalized manner appeared to promote QoL. Interaction formats (individual, group, remote), session duration [range=1.5-28] and number of sessions [range=1-18] varied between the studies reviewed.

**Conclusion:**

Thoughtful, structured design of educational programs requires a match between the educational aspects specific to each individual and the appropriate choice of content, delivery modalities of the interventions and evaluation protocol, as well as a reasonable follow-up time. The conclusions drawn could serve as guidelines to direct future research towards optimal educational interventions.

**Systematic Review Registration:**

PROSPERO CRD42022338651.

## Introduction

 Multiple sclerosis (MS) is a condition characterized by demyelination in the central nervous system^1^ affecting over 2.8 million people worldwide.^2^ It is often diagnosed at an early age, generally between 20 and 40 years. The condition is more prevalent in women,^1^ and remains the primary cause of substantial neurological disability.^3^ Its progressive, chronic and unpredictable nature^4^ is generally associated with a number of fluctuating symptoms such as ataxia, cognitive and vision problems, fatigue, sexual dysfunction and urinary and intestinal problems.^1,5^ At the same time, these symptoms are accompanied by depressive disorders and anxiety. All aspects of the MS patient’s daily life are accompanied by intense uncertainty, which prolongs their adaptation period.^6^ The unpredictable course of the disease has pronounced physical, psychological and social repercussions.^4^ As a result, quality of life (QoL) in MS is estimated to be lower than in the general population.^7^ Patients face a considerable challenge in maintaining their autonomy.^8^

 As with many other chronic diseases, it is essential to ensure continuous, integrated and coordinated management for people with multiple sclerosis (pwMS) This includes adherence to treatment, symptom management and strategies to overcome acute exacerbations.^9^ The various challenges encountered during the course of MS require the active involvement of the individual and their caretaker in the care pathway^10^ within the framework of a biopsychosocial perspective.^11^ Given this requirement, therapeutic approaches are currently opening up to non-pharmacological approaches.^12^ In this respect, therapeutic education (TPE) is experiencing remarkable growth as an essential non-pharmacological intervention^13^ in the management of people with chronic diseases, including MS.^8^ According to the 1998 WHO definition, TPE is defined as a continuous process which improves the individual’s understanding of the disease, the management of symptoms, and the acquisition or maintenance of self-care and coping skills. This is achieved through structured activities and psychological support aimed at informing the patient about the disease, health and care-related behaviors, and the functioning of hospital facilities.^14^

 Several reviews have provided evidence on the success of TPE programs in the management of MS, with improvements in outcomes, such as treatment adherence, depression, anxiety and fatigue.^15-18^ Thanks to the new skills incorporated in TPE programs, a positive change in behavior has been established.^19^ These programs not only improve QoL but also empower the individual to become more autonomous^19^ and an expert of their disease.^20^

 However, there is considerable variability in the effectiveness of TPE programs for pwMS in terms of content and delivery modalities,^17^ given the lack of a standardized conceptual framework.^8^ In general, the implementation of educational programs is considered a complex endeavor^17,21^ due to an often incoherent description and a lack of clarification of the key components likely to improve their evaluation.^21^ Indeed, a meta-analysis examined lifestyle self-management regimes in pwMS,^22^ and demonstrated that the effectiveness of the dimensions identified on well-being is questionable given the heterogeneity between the included studies which limited the possibility of reliably pooling their effects. Another review highlighted the need to focus on MS educational programs that use directly relevant outcomes to the disease, such as QoL.^9^ As such, there is a pressing need for a thorough understanding of TPE programs in order to optimally target an appropriate design within a structured framework. With this in mind, a systematic review was undertaken; the first to our knowledge. Its aim was to synthesize the impact of TPE programs on the QoL of pwMS, by describing the various constituent elements of these programs (structure, content and delivery modalities) and their interactions, which are likely to influence their effectiveness. The results will enable researchers to transcend the difficulties associated with appropriating educational programs, thereby promoting the consistent acquisition of the coping and self-management skills needed to optimize the individual’s QoL.

## Materials and Methods

 Following a pre-established protocol^23^ that has been registered in the Prospective International Register of Systematic Reviews (PROSPERO, CRD42022338651), this systematic review was conducted following the PRISMA (Preferred Reporting Items for Systematic Reviews and Meta-Analyses) guidelines.^24^

###  Search strategy

 To identify relevant articles, a targeted search was performed in the PubMed, Web of Science and Scopus databases for articles published between 2007 and 2022. Keywords “Multiple Sclerosis”, “Therapeutic Education” and “Quality of Life” were used together with Mesh terms (Medical Subject Headings), Boolean logical operators (“AND” and “OR”) and appropriate truncation. That way, a specific search strategy was adjusted according to the mapping of terms in each database** (**Supplementary file 1**)**

###  Eligibility criteria

 The criteria for including and excluding studies were determined based on the PICOS model (Participants, Intervention, Comparison, Outcome, and Study design type) (Table 1).

**Table 1:** PICOS eligibility criteria

	**Inclusion criteria**	**Exclusion criteria**
Population	Participants > 18 years old with a diagnosis of definite MS with no form restrictions	
Intervention	TPE program is defined as any structured intervention which mobilizes or maintains the patient's skills. It includes learning, transmission of information related to the disease and education on self-management strategies.	TPE intervention judged not to be explicit in terms of its objective and content.
Comparison	Studies, whether they had a control group or not, were eligible for inclusion.	
Outcomes	QoL was presented as a primary/secondary outcome and measured at two or more time points (baseline, post-intervention, follow-up), and assessed by a valid instrument	Studies that did not assess QoL
Type of studies	All interventional and observational studies were included, with no restrictions on the language of publication.	Qualitative, pilot study, abstract, research protocol, dissertation

###  Study selection

 A two-stage process was used to filter articles for inclusion in this review. First, after removing duplicates, two researchers (IR and IE) individually reviewed the titles and abstracts of publications retrieved in the original search, excluding studies that did not meet the inclusion criteria. Second, the same researchers assessed the full-text of the articles to determine whether they met the criteria for inclusion in the review. Any inconsistencies between the reviewers were resolved by discussion and consensus, and any disagreements were resolved by consulting with a third researcher (MER).

###  Data extraction

 Using a standardized form designed specifically for this review, the two reviewers (IR and IE) independently extracted all pertinent data from the included studies. This form was first pilot-tested to verify it and make any necessary modifications. The information was extracted and grouped in two separate tables, including information on:

 S*tudy characteristics: *Author, country, year of publication, sample (size, sex, age of participants, form of MS and Expanded Disability Status Scale [EDSS]^25^), study design, tools and number of QoL measures, duration of total follow-up and summary of main results.

 C*haracteristics of TPE programs*: The systems approach to TPE^14^ was used to frame and structure the content and delivery modalities of educational programs. This conceptual framework plays a crucial role in the design and effective implementation of TPE programs.^8,14,19^ The structuring of the programs was based on the different stages of TPE.^12,26^ The first stage involves the development of an educational diagnosis, followed by the definition of a personalized education program. The second stage includes implementing the educational sessions, while the final stage involves assessing the patient’s skills. The content and delivery modalities of the programs are integral parts of the implementation stage. The extraction of program content took into account several elements: the type of the underlying approach/model/theory, the skills taught, in particular cognitive (knowledge, decision-making and reasoning), sensorimotor and psycho-affective skills.^12^ For delivery modalities, the authors identified the teaching methods, mode of interaction (individual, group or distance), duration and frequency of educational sessions, program facilitator, educational support sessions and involvement of caregivers.

###  Quality assessment 

 The quality of the studies was assessed by the two reviewers (IR and IE) to evaluate the methodological quality and relevance of the eligible articles. For this, tools were deployed according to each study’s design.^27^ The Cochrane Risk of Bias (RoB) 2.0 tool was employed for randomized controlled trials (RCTs),^28^ the ROBINS-I (Risk Of Bias In Non-randomised Studies-of Interventions) tool for quasi-experimental studies,^29^ the National Institute of Health (NIH) quality assessment tool for before/after trials (Pre-Post) without a control group,^30^ and the Joanna Briggs Institute (JBI) critical appraisal checklist for cross-sectional studies.^31^

###  Data synthesis 

 Given the heterogeneity attested in the studies included in this review (designs, study populations, intervention components, follow-up periods and judgment criteria), a meta-analysis of the available evidence was not possible. Consequently, based on the established summary tables, a qualitative and narrative approach was used to identify the structure and characteristics of TPE programs (content and delivery modalities) likely to produce a significant change (*P* < 0.05) in QoL outcomes.

## Results

**Figure 1:**
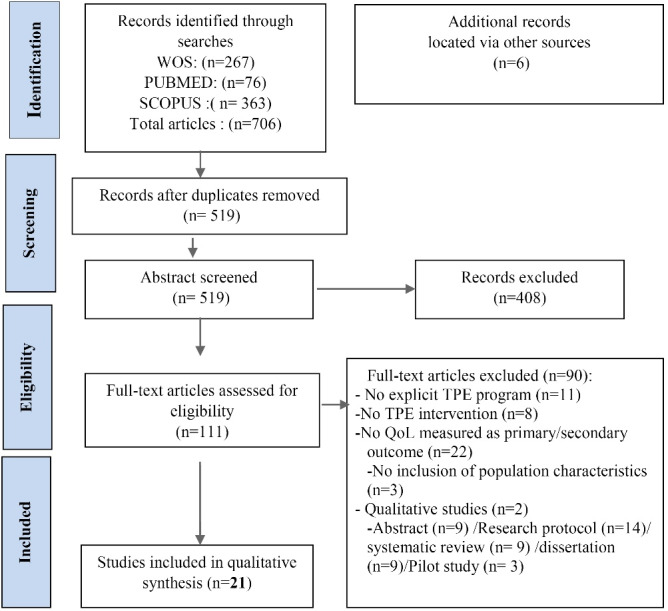
PRISMA diagram of the study selection process

 A total of 706 articles were identified through database searching, and four publications were identified through other sources. After excluding duplicates, the titles and abstracts of 519 studies were reviewed for relevance. Of the 111 articles retrieved for further full-text assessment, 90 were excluded for not meeting the eligibility criteria. At the end, 21 articles were retained for this review.Figure 1 presents the PRISMA flowchart depicting the study selection process.

###  Study characteristics

 The characteristics of the included studies are presented in Table 2. Studies came from different countries, including the United States (n = 6), Germany (n = 4), the United Kingdom (n = 3), Italy (n = 2), Iran (n = 2), New Zealand (n = 1), Turkey (n = 1), France (n = 1) and Australia (n = 1). These studies used the following designs: RCTs (n = 14), quasi-experimental studies (n = 2), pre-post trials without a control group (n = 3) and observational studies (n = 2). Sample size in the selected studies ranged from 24 to 275 participants, with a total number of 2495 participants, aged between 30 and 56 years (M_age_ = 47.84 years). The majority of participants were women (79.3%). In the 15 studies reporting the type of MS, the most frequent was relapsing-remitting MS (73.42%). Only eight studies included EDSS scores, showing minimal to moderate disability. QoL was measured as a primary outcome in 15 studies, and the instruments used to assess outcomes varied between studies. The most commonly used tools to assess changes in QoL were the Short Form Health Survey (SF_36_) or its abbreviations (SF_8_ or SF_12_) (n = 8), the Multiple Sclerosis Quality of Life-54 (MSQOL-54) (n = 4) and the Hamburg QoL Questionnaire (HAQUAMS) (n = 4).

**Table 2:** Study characteristics

**First author year, country**	**Study design**	**Sample size ** **Age (Mean±SD)**	**Gender females** **[%]**	**MS type**	**EDSS**	**Outcomes (Measure)**	**Number of QOL measurements**	**Follow-up**	**Results**
Oz et al^41^ (2020), Turkey	RCT	80 (41.5 ± 10.6)	75.5%	NR	3.07 ± 2.05	MSQOL-54	T1: Before T2: After T3: 3 months	3 months	Significant improvement in QOL in the treatment group after 3 months (*P* < 0.05)
Köpke ‎et al^43^(2009), Germany	RCT	150 (37.3 ± 7.2)	82%	RRMS	NR	HAQUAMS	T1: Before T2: 2 years	2 years	No significant change in QOL between groups
Köpke et al^42^(2014), Germany	RCT	192 (36.5 ± 10.3)	74%	RRMS	NR	HAQUAMS	T1: 2 weeks before T2: 2 weeks after T3:12 months	12 months	No significant change in QOL between groups
Miller et al^52^ (2011), USA	RCT	167 (48.1 ± 9.1)	72%	NR	NR	EURO-QOL5	T1: Before T2:12 months	12 months	No significant change QOL between groups
Mulligan et al^48^(2016), New Zealand	Pre-Post -without a control group	24 (49.29 ± 8.12)	100%	RRMS SPMS PPMS	NR	SF12	T1: One month before T2: Immediately T3: After 6 months	6 months	No significant change in QOL between groups
Thomas et al^40^ (2013), UK	RCT	146 (48.0 ± 10.2)	73%	Benign MS RRMS SPMS PRMS	NR	MSIS-29 QALYs SF-36v2	T1: 1week before T2: 1 month T3: 4 months	4 months	No significant difference in QOL for treatment group except for vitality subscale
Brittle et al^37^(2008), UK	Observational	105 (48 ± 11.56)	81.2%	NR	NR	MSQOL-54	T1: Before T2: After	10 weeks	No significant difference in QOL for treatment group
Mathiowetz et al^47^(2007), USA	RCT	169 (48.34 ± 8.44)	82.8%	RRMS SPMS PPMS PRMS	NR	SF36	T1: 1week after T2: 7 weeks T3: 13weeks T4: 65weeks	1 year	Significant improvement in 4 out of 8 QOL subscales in the treatment group (*P* < 0.05) with one-year maintenance
Feicke et al^38^(2014), Germany	Quasi experimental	64 (41.94 ± 11.71)	87.1%	RRMS SPMS PPMS	NR	HAQUAMS	T1: Before T2: Post T3: 6 months	6 months	Significant improvement in QOL in the intervention group (*P* < 0.05)
Seifi et al^32^(2018), Iran	Pre-Post without a control group	28 60.7% (age under 40)	64.3%	NR	NR	WHOQOL-BREF	T1: Before T2: After	4 weeks	Significant improvement of the 4 QOL items in the treatment group (*P* < 0.05)
Momenabadi et al^51^(2020), Iran	RCT	80 (30.43 ± 3.8)	87.5%	RRMS	< 5	MSQOL-54	T1: Before T2: One week after T3: After 2 months	2 months	Significant improvement in QOL in physical function treatment group maintained at two months after (*P* < 0.05)
Plow et al^39^(2019), USA	RCT	208 (53.2 ± 6.5, 51.2 ± 9.2)	90% 79.9%	RRMS SPMS PPMS	NR	MSIS	T1: 2 weeks before T2: 14 weeks T3: 26 weeks	26 weeks	No statistically significant difference in the 2 components of QOL for treatment group
Finlayson et al^44^(2011), USA	RCT	190 (56 ± 9)	79%	RRMS SPMS PPMS PRMS	NR	SF36	T1: Before T2: immediately after T3: After 6 weeks T4: 3 months T5: 6 months	6 months	Significant improvements in 6 of the 8 QOL subscales in the treatment group (*P* < 0.05) maintained at 6 months.
Gallien et al^34^ (2020), France	Observational study	29 (41.1 ± 9.5)	86.20%	NR	< 3: 1.7 (1.1)	SF36	T1: Before T2: 6 months	6 months	Significant improvement in MCS in treatment group (*P* < 0.05) maintained at 6 months
Ehde et al^35^ (2015), USA	RCT	163 (51.0 ± 10.1)	89.3%	RRMS PPMS	< 4.0: (25.3) 4.5-6.5: (61.3) > 7.0: (13.3)	SF8	T1: Before T2: 9 and 11 weeks after T3: 6 Months T4:12 Months	12 months	Significant improvement in QOL in both groups, maintained at 6 and 12 months (*P* < 0.05)
Graziano et al^46^(2014), Italy	RCT	82 (42.3 ± 8.5)	66%	RRMS PPMS PSMS	Between 1 and 5.5	MSQOL-54	T1: Before T2: Just after T3: 6 Months	6 months	Significant improvement in QOL in the group intervention maintained at 6 months (*P* < 0.05)
Calandri, et al^45^ (2017), Italy	Quasi experimental design	85 (38 ± 12.5)	61%	RRMS PPMS SPMS	Between 1 and 4	SF12	T1: Before T2: 6 months T3: 1 year	1 year	MCS QOL improved significantly in the intervention group and was maintained at one year.
Ghahari et al^50^(2010), Australia	RCT	95 (51 ± 13.6, 47.86 ± 12)	91.2%	RRMS PPMS SPMS	NR	PWI	T1: Before T2: After T3: 3 Months	3months	Significant improvement in QOL in treatment group at posttest (*P* < 0.05)
Hartely et al^33^(2009), UK	Pre-Post -without a control group	33 49 (20–74)	82%	NR	< 6	LMSQOL	T1: Before T2: After	14 weeks	Significant improvement in QOL in treatment group (*P* < 0.05)
Bombardier et al^36^ (2008), USA	RCT	130 47.5 (41 to 54)	75.7%	RRMS	< 5.5	SF-36	T1: Before T2: 12 weeks	12 weeks	Significant improvement in MCS in treatment group (*P* < 0.05)
Pöttgen et al^49^ (2018), Germany	RCT	275 40.80 (11.1)	82%	RRMS SPMS PPMS	NR	HAQUAMS	T1: Before T2: 12 weeks T3: 24 weeks	3 months	Significant improvement in QOL in the treatment group in 3 scales maintained at 3 months (*P* < 0.05)

**Abbreviations: **QOL, quality of life; MS, multiple sclerosis; RCT, randomized controlled trial; MCS, mental composite score; PCS, physical composite score; EDSS, Expanded Disability Status Scale; (I), Intervention; (C), Control; RR, relapsing-remitting; SP, secondary progressive; PP, primary progressive; PR, progressive relapsing; QALYs, quality of adjusted life years; SF-8-12-36, Short Form 8-12-36; HAQUAMS, Hamburg quality of life questionnaire; MSIS, Multiple Sclerosis Impact Scale; MSIS_29_, Multiple Sclerosis Impact Scale-29; MSQOL-54, Multiple Sclerosis Quality of Life-54; WHOQOL-B, World Health Organization Quality of Life; EURO-QOL5, Euro quality of life with 5 dimensions of health; LMSQOL, Leeds Multiple Sclerosis Quality of Life; PWI, Personal Wellbeing Index.

###  Characteristics of TPE programs

 There was considerable variation between programs in terms of structure, content and delivery modalities (Table 3).

**Table 3:** Characteristics of TPE programs

**First author** **year,** **country**	**Educational diagnosis**	**Personalized program**					**Program implementation**			**Evaluation**
			**Approach/** **model/Theory underlying**	**Skills taught**					**Interaction+Duration (week)/Sessions (n), total duration [h]+Facilitator+Teaching Method**	
				**CS**			**SS**	**Psy. S**		
				**K**	**D**	**R**				
Oz et al^41^ (2020), Turkey			CBT	•		•		•	Group (F/F) 8 wk, 4 S, 8 h Researchers Interactive presentation + discussion	•
Köpke ‎et al^43^(2009), Germany			Theory of protective motivation	•	•			•	Group (F/F) 1 wk, 4 h MS nurse + trained patient Interactive presentation + discussion + educational booklet	•
Köpke et al^42^(2014), Germany			Theory of planned behavior	•	•			•	Group (F/F) 1 S, 4 h Non-medical staff Interactive presentation + discussion, brochures	•
Miller et al^52^(2011), USA			SM strategies + self-monitoring strategies	•					Remote (Online) NR No instructor Self-learning Online + discussion	
Mulligan et al^48^(2016), New Zealand			SM strategies	•	•	•			Group (F/F) 6 wk, 6 S, 12 h Phy + OT Interactive discussion + case studies + reflexion form	•
Thomas et al^40^(2013),UK			CBT + socio-cognitive, energy efficiency + SM + SE	•				•	Group (F/F) 6 wk, 6 S, 9 h MS nurse + Phy + OT Interactive presentation + discussion	•
Brittle et al^37^(2008), UK	•	•	Conductive education approach	•			•		Group (F/F) 10 wk, 10 S, 15 h to 20 h Care manager Demonstration	
Mathiowetz et al^47^(2007),USA			Energy conservation psychoeducational theory	•			•		Group (F/F) 6 wk, 6 S, 12 h OT Interactive Presentation + discussion	•
Feicke et al^38^(2014), Germany	•		SM strategies	•				•	Group (F/F) 5S, 7h Neurologist + MS nurse + Psy Interactive discussion + Mind maps	
Seifi et al^32^(2018), Iran	•		Self-care behavior system model	•			•		Group (F/F) 2 S, 1.5 h NR NR	•
Momenabadi et al^51^(2020), Iran			Self-care behavior system model + SE.	•	•	•	•	•	Group (F/F) + Remote (telephone, Online) 12 wk, 18 S, 13.5 h to 18 h NR Brainstorming + Interactive discussion + Reading	•
Plow et al^39^(2019), USA			SM strategies + Energy conservation	•			•	•	Remote (telephone) 12 wk, 6 S OT + Research assistant NR	
Finlayson et al^44^(2011), USA			SM strategies	•	•	•	•	•	Remote ( telephone) 6 wk, 6 S, 7 h OT Interactive discussion + Learning manual	•
Gallien et al^34^(2020), France	•	•	SM strategies	•			•		Group (F/F) 1 day Care manager Role-playing + Picture expression + Support sessions	
Ehde et al^35^(2015),USA	•		CBT + SM + evidence-based positive psychology	•	•	•	•	•	Remote (telephone) 8 wk, 8 S, 6 h to 8 h SW + Psy Interactive discussion + Case studies + Support sessions	•
Graziano et al^46^(2014), Italy			CBT	•			•	•	Group (F/F) 8 wk, 5 S, 10 h Psy Interactive discussion + demonstration + support sessions	•
Calandri, et al^45^ (2017), Italy			CBT	•			•	•	Group (F/F) 8 wk,6S, 12h Psy Interactive discussion + demonstration + Support sessions	•
Ghahari et al^50^(2010), Australia			SE + SM strategies	•	•	•		•	Remote (Online) 7 wk, 7 S, 14 h to 21 h OT Interactive discussion + demonstration	
Hartely et al^33^(2009), UK	•	•	SM strategies + exercise	•			•	•	Group (F/F) 14 wk, 14 S, 28 h Phy + Psy + MS nurse + Neurologists Brainstorming + Demonstration + Support sessions	
Bombardier et al^36^ (2008), USA	•		Motivational interviewing	•			•	•	Individual (F/F) + Remote (telephone) 6 S, 9 h to 12 h Care manager Interview telephone advice + support sessions	
Pöttgen et al^49^ (2018), Germany			CBT + related psychotherapeutic approaches	•			•	•	Remote (Online) 2 times/wk No instructor Simulated dialogue	

Abbreviations: MS, multiple sclerosis; SW, social worker; SM, self-management; SE, self-efficacy; CBT, cognitive behavioral therapy; OT, occupational therapist; SC, cognitive skills; S. Psy, psychoaffective Skills; SS, sensorimotor skills; K, knowledge; D, decision-making; R, Reasoning; NR, not reported; Psy, psychologist; Phy, physiotherapist; h, hour; F/F, face to face; wk, week; S, Sessions; • = Present; Blank = Not present

*Program structure*: The four stages of TPE were presented in the programs as follows: development of an educational diagnosis (n = 7),^32-38^ personalization of the educational program (n = 3),^34,36,37,39^ implementation of the program (n = 21) and evaluation of the skills acquired (n = 11).^32,35,40-48^

*Program content*: The TPE programs were based on several underlying theories, models or approaches, such as the cognitive behavioral therapy (CBT) model (n = 6),^35,40,41,45,46,49^ the self-efficacy theory (n = 3),^40,50,51^ the conservation of energy approach (n = 3),^39,40,47^ the system model of self-care behaviors (n = 2),^32,51^ and the self-management approach which can be implemented independently (n = 5),^34,38,44,48,52^ or guided by the theories/models previously mentioned (n = 4). With regard to the skills targeted, all the studies focused on cognitive skills, in particular the development of the patient’s knowledge of MS (n = 21), seven studies targeted decision-making,^35,42–44,48,50,51^ and six intervention studies focused on reasoning skills.^35,41,44,48,50,51^ In addition, sensorimotor skills were targeted in 13 programs,^32-37,39,44-47,49,51^ while psycho-affective skills developed in 15 studies.^33,35,36,38-46,49-51^

*Program delivery modalities*: Group sessions with face-to-face interaction were the most commonly used (62%).^32-34,37,38,40-43,45-48^ In 28% of the studies, no direct communication with the educational program facilitators was included, either remotely by telephone (n = 3)^35,39,44^ or online (n = 3).^49,50,52^ Finally, two interventions combined remote interaction with a group modality or with an individual modality. The professionals involved in running the programs were mainly occupational therapists (n = 6), psychologists (n = 5), nurses (n = 4) and physiotherapists (n = 3). Only one study^43^ involved a trained expert patient. The shortest intervention lasted for 1.5 hours,^32^ while the longest lasted for 28 hours.^33^ The average duration of the sessions was 10.07 hours. The number of educational sessions varied between 1 and 18, with a median value of 6. Of the 21 interventions studied, five were short ( < 7 hours),^32,35,38,43,44^ eight were medium (7-15.75 hours)^36,40,41,45–48,51^ and three were long ( > 15.75 hours).^33,37,50^ Follow-up ranged from 4 weeks to 24 months after the intervention. As for the skills taught, they were put into practice through an interactive approach in the majority of the programs (n = 18), through methods such as brainstorming, simulated dialogue, case studies, role-playing, interactive presentations and discussions. In addition, only six interventions^33-36,45,46^ proposed sessions of educational recall after the end of the formal educational program and two others involved caregivers in the implementation of the programs.^37,49^

###  Effects of the programs on QoL

 A positive effect of TPE programs showing statistically significant differences in QoL (*P* < 0.05) was observed in 14 studies (67%)^32-36,38,41,44-47,49-51^ This improvement was maintained at follow-up in 42% of the studies.^34,35,41,44–47,49,51^ These results were observed in patients with minimal to moderate disability according to the EDSS score. A percentage of 33% of the included studies indicated no effect on QoL at any of the assessed time points. QoL was measured using multidimensional questionnaires, and the tool most commonly used to assess change was the SF_36_ or its abbreviations. Its physical or mental components were measured in 62% of the studies,^32,34-37,39-41,44,45,47,48,51^ while 38% of the studies reported scores on the QoL subscales. The mental component significantly improved in 9 out of 12 of the interventions (75%),^32,34-36,38,41,44,45,47^ which exceeded the physical component, only improving in 6out of 12 of the studies (50%).Moreover, these two components were simultaneously developed in five programs. According to the TPE systemic approach, promotion of QoL was associated with 6 out of 7 of the interventions (85%)^32-36,38^ which began with an initial educational diagnosis, as well as with 2 out of 3 of the studies (67%) using a personalized program^34,36^ and with 8 out of 12 of the programs (67%)^32,35,41,44-47^ evaluating the objectives taught. This positive effect was also observed in 10 out of 13 of the studies (72%)^33,35,36,41,44-46,49-51^ which had as their common objective the development of cognitive, psycho-affective and sensorimotor skills. An improvement in QoL was also noted in 5 out of 6 interventions (83%) which incorporated the CBT approach,^35,41,45,46,49^ and in only one study out of 3 which used energy conservation strategies.^47^ This was also true for 60% of the interventions incorporating self-management principles in isolation (n = 3).^34,38,44^ In addition, of the 13 programs based on group interaction, a significant proportion, around 61%, showed positive results in terms of QoL using interactive methods. The same finding was observed in 4 out of 6 programs while using distance modalities (online: n = 2; telephone: n = 2).In general, programs with long educational sessions ( > 15.75 hours) also showed a significant effect on QoL in 2 out of 3 of the studies (67%).^33,50^ This significant change was also observed in all programs that scheduled educational support sessions after the intervention.

###  Quality of studies

 The Cochrane RoB 2.0 tool^28^ was applied to evaluate the methodological quality of 13 RCTs. In terms of overall bias, six studies had some concerns,^36,41,46,47,50,52^ six had low risk,^35,39,43,44,49,51^ while two had high risk as they did not report outcomes of QoL.^40,42^Low and moderate risks were also found respectively for two experimental studies^38,45^using the ROBINS-I tool.^29^ High risk was modulated by participant selection bias and other confounding factors. Using the NIH assessment tool,^30^ one study was of good quality with a score of over 80% positive responses,^32^ and two others were judged to be of average quality with a score of 60%.^33,48^ Two other studies were assessed using the JBI^31^ tool for cross-sectional studies. One study was considered of medium quality with a total score of 62% positive responses,^37^ while the other was considered of poor quality with a score of 37% due to a lack of clarity regarding sample inclusion criteria, statistical analysis and confounding factors^34^**(**Supplementary file 2).

## Discussion

 In this systematic review, the characteristics of 21 TPE programs and their effectiveness on QoL were synthesized. Overall, the results showed that the majority of programs appear to have a positive impact on QoL. However, it should be noted that the programs are multimodal, but are incomplete in their design due to a lack of detail.^53^ Complexity and heterogeneity have been noted in the content and delivery modalities. This limits both the feasibility of a comprehensive evaluation and the ability to replicate an evidence-based educational intervention.^54^

 Indeed, a clear improvement in QoL accompanied the educational programs in the study that began with the first two stages of the TPE approach: educational diagnosis and the establishment of a personalized plan. However, the inclusion of participants was generally limited to registers and databases without taking into account their specific needs. TPE programs must be tailored to each patient’s symptoms^8^; otherwise, the educational program will fail.^12^ Adequate training is essential to enable healthcare professionals to develop patient-centered programs which encompass a wider range of elements, such as cognitive, psychological, social and cultural aspects^55^ in order to offer personalized management.^56^ In the implementation phase, programs that incorporate CBT approaches as a psychological intervention^57,58^ and those that adopt its components such as self-management^59^ are closely linked to promotion of QoL.^60,61^ CBT therefore complements TPE in an effective way.^62^ In general, programs based on behavioral interventions are a key component in enabling patients to live well with MS.^59^ However, they are supported by a limited number of studies, hence the need for further research. Another aspect that arises is that the studies included do not provide sufficient theoretical justification to facilitate comparison between different programs. It is therefore essential that these programs are optimally designed on the basis of a clear theoretical framework; otherwise, their effectiveness and reliability will be affected.^63^ This framework must take into account the social determinants of the individual as well as his/her physical and mental state.^64^ The broad spectrum of unpredictable and fluctuating symptoms from which MS patients suffer^1,3^ requires researchers to integrate a variety of skills when designing educational programs. In other words, simply teaching knowledge is not enough to change all lifestyle habits. It is necessary to develop other skills, such as problem-solving, sensorimotor and psycho-affective skills, which contribute to maintaining a high QoL.^17^ Thus, the completion of the skills assessment stage remains the only determinant that provides professionals with information on the extent to which patients have achieved the educational, clinical or therapeutic objectives of the programs.

 Furthermore, the results showed that programs delivered using a participatory interactive method over a long period of time ( > 15.75 hours) produced statistically significant differences in QoL compared to short-term interventions. The duration and dosage of the educational sessions may be a determinant of effectiveness.^18^ Although the majority of the programs in the study delivered in a group setting (face-to-face) appeared to be cost-effective and to have promising effects, the difficulty of organizing TPE groups is linked to the homogeneity of the participants. This homogeneity is conditioned by compatibility with the organizational conditions, needs and common characteristics of the participants (pace, learning style, etc.). Information communication technologies can be deployed to improve access to available services by overcoming the constraints linked to MS comorbidities such as fatigue and disability, as well as distance and travel costs.^65-67^ It is legitimate to objectively explore educational techniques (telephone, email, mobile applications, etc) enabling individuals and their families to positively manage their own care at a distance if face-to-face activities are restrictive.^9,12^

 Although a positive effect of QoL was maintained at follow-up in 46% of the studies, there were studies with short follow-up periods that did not allow sufficient time for the benefits of behavioral change to occur. Positive changes in habits and behavior therefore depend on the time factor for optimal integration of skills into daily life.^18^ This evolution also requires continuous and dynamic education in the form of feedback sessions and updates of the initial educational situation.^56^ In line with the results of this study, promotion of QoL was observed following regular visits or contacts after the intervention.

 Caregivers (the family) are involved in the care process. However, few of the TPE programs integrated them to develop specific skills. Caregivers find themselves obliged to devote more time, emotions and physical activity. It is at this point that their own needs are hardly taken into consideration.^68,69^ Their QoL can consequently be reduced.^70,71^ Future research must take into account the active involvement of caregivers by proposing educational programs that promote their QoL alongside their sick relatives. Also, peers should also be involved, as their active participation fosters the learning process through sharing experiences^72^ and promoting the QoL and self-efficacy of MS patients.^73^

## Limitations

 In this review, the comparison between various TPE programs posed a challenge due to the substantial heterogeneity between studies. This diversity was primarily influenced by the inherent complexity of TPE program characteristics, different outcome dimensions and variability in study design. It also stems from differences in the sensitivity of the tools used to assess changes resulting from interventions, given that only nine studies used an instrument specifically measuring the QoL of MS patients. The results therefore should be interpreted in light of this limitation. In addition, some studies restricted their sample to individuals with relapsing-remitting MS, which reduces the possibility of generalizing these results to other disease phenotypes, such as progressive MS. Other limitations of the study include the terminological complexity of the concept of TPE. The diversity of interpretations of this term in the literature may lead to variability in the selection of relevant studies, which could influence the representativeness of the results. In addition, the choice of only three databases for this review and the absence of a grey literature search restricted the number of studies included. A wider selection would have provided a broader perspective on the impact of TPE programs on the QoL of pwMS.

## Conclusion

 TPE programs appear to have a positive impact on the QoL of pwMS. Educational programs that focus on the individual needs of patients and aim to develop their skills in a personalized way are promising. The key to the success of a TPE program a well thought-out and structured design. This design requires a match between the actual educational aspects of the patient and the appropriate choice of content, delivery modalities of the interventions and evaluation protocol, as well as a reasonable follow-up time. In this respect, it is imperative to standardize a specific methodology, based on a structured framework^8^ in order to simplify the description of TPE programs and establish common criteria for evaluating and comparing their effectiveness. Despite the encouraging results of this study, the current evidence is insufficient to make sound recommendations. The conclusions drawn can be used as basic guidelines to direct future research towards optimal educational intervention.

## Acknowledgments

 We are grateful to all researchers whose articles were reviewed in the study.

## Competing Interests

 The authors declare that they have no competing interests.

## Ethical Approval

 Not applicable.

## Supplementary Files


Supplementary file 1: contains the search strategy

Supplmentary file 2: contains the quality of studies

## References

[1] Noseworthy JH, Lucchinetti C, Rodriguez M, Weinshenker BG (2000). Multiple sclerosis. N Engl J Med.

[2] MS International Federation. Atlas of MS 3rd Edition, PART 1: Mapping Multiple Sclerosis Around the World Key Epidemiology Findings. 2020. Available from: https://scleroseenplaques.ca/ressources/nouvelles/article/latlas-de-la-sp-presente-les-principaux-facteurs-faisant-obstacle-a-une-prise-en-charge-clinique-efficace-de-la-sclerose-en-plaques-dans-le-monde. Accessed January 13, 2023.

[3] Pérennes M, Ollivier C, Lorillon P (2008). La sclérose en plaques, première cause non traumatique de handicap sévère acquis. Actual Pharm Hosp.

[4] Pagnini F, Bosma CM, Phillips D, Langer E (2014). Symptom changes in multiple sclerosis following psychological interventions: a systematic review. BMC Neurol.

[5] Comabella M, Khoury SJ (2012). Immunopathogenesis of multiple sclerosis. Clin Immunol.

[6] Simmons RD (2010). Life issues in multiple sclerosis. Nat Rev Neurol.

[7] Peters S, Wilkinson A, Mulligan H (2019). Views of healthcare professionals on training for and delivery of a fatigue self-management program for persons with multiple sclerosis. Disabil Rehabil.

[8] Demaille-Wlodyka S, Donze C, Givron P, Gallien P (2011). Self care programs and multiple sclerosis: physical therapeutics treatment - literature review. Ann Phys Rehabil Med.

[9] Rae-Grant AD, Turner AP, Sloan A, Miller D, Hunziker J, Haselkorn JK (2011). Self-management in neurological disorders: systematic review of the literature and potential interventions in multiple sclerosis care. J Rehabil Res Dev.

[10] Carton H, Loos R, Pacolet J, Versieck K, Vlietinck R (1998). Utilisation and cost of professional care and assistance according to disability of patients with multiple sclerosis in Flanders (Belgium). J Neurol Neurosurg Psychiatry.

[11] Wagner EH, Bennett SM, Austin BT, Greene SM, Schaefer JK, Vonkorff M (2005). Finding common ground: patient-centeredness and evidence-based chronic illness care. J Altern Complement Med.

[12] d’Ivernois JF, Gagnayre R. Apprendre à Éduquer le Patient: Approche Pédagogique. 3rd ed. Paris, France: Editions Maloine; 2008.

[13] Mackay AM, Buckingham R, Schwartz RS, Hodgkinson S, Beran RG, Cordato DJ (2015). The effect of biofeedback as a psychological intervention in multiple sclerosis: a randomized controlled study. Int J MS Care.

[14] World Health Organization (WHO). Therapeutic Patient Education: Continuing Education Programmes for Health Care Providers in the Field of Prevention of Chronic Diseases: Report of a WHO Working Group. Copenhagen: WHO Regional Office for Europe; 1998. Available from: https://iris.who.int/handle/10665/108151. Accessed March 23, 2023.

[15] Wendebourg MJ, Heesen C, Finlayson M, Meyer B, Pöttgen J, Köpke S (2017). Patient education for people with multiple sclerosis-associated fatigue: a systematic review. PLoS One.

[16] Kidd T, Carey N, Mold F, Westwood S, Miklaucich M, Konstantara E (2017). A systematic review of the effectiveness of self-management interventions in people with multiple sclerosis at improving depression, anxiety and quality of life. PLoS One.

[17] Plow MA, Finlayson M, Rezac M (2011). A scoping review of self-management interventions for adults with multiple sclerosis. PM R.

[18] Hersche R, Roser K, Weise A, Michel G, Barbero M (2022). Fatigue self-management education in persons with disease-related fatigue: A comprehensive review of the effectiveness on fatigue and quality of life. Patient Educ Couns.

[19] Grange L, Allenet B (2013). [Principle and practical implementation of the therapeutic patient education (TPE)]. Rev du Rhum Monogr.

[20] Bodenheimer T, Lorig K, Holman H, Grumbach K (2002). Patient self-management of chronic disease in primary care. JAMA.

[21] Craig P, Dieppe P, Macintyre S, Michie S, Nazareth I, Petticrew M (2008). Developing and evaluating complex interventions: the new Medical Research Council guidance. BMJ.

[22] Wills OC, Probst YC (2022). Understanding lifestyle self-management regimens that improve the life quality of people living with multiple sclerosis: a systematic review and meta-analysis. Health Qual Life Outcomes.

[23] Raji I, El Harch I, Ragala MEA, Berraho M, Belahsen MF (2023). The impact of therapeutic education programs on the quality of life of patients with multiple sclerosis: protocol of a systematic review. J Educ Health Promot.

[24] Moher D, Liberati A, Tetzlaff J, Altman DG (2009). Preferred reporting items for systematic reviews and meta-analyses: the PRISMA Statement. Open Med.

[25] Kurtzke JF (1983). Rating neurologic impairment in multiple sclerosis: an Expanded Disability Status Scale (EDSS). Neurology.

[26] Haute Autorité de Santé. Sclérose en plaques. 2006. Available from: https://www.has-sante.fr/jcms/c_460315/fr/ald-n-25-sclerose-en-plaques. Accessed October 5, 2022.

[27] Ma LL, Wang YY, Yang ZH, Huang D, Weng H, Zeng XT (2020). Methodological quality (risk of bias) assessment tools for primary and secondary medical studies: what are they and which is better?. Mil Med Res.

[28] Sterne JA, Savović J, Page MJ, Elbers RG, Blencowe NS, Boutron I (2019). RoB 2: a revised tool for assessing risk of bias in randomised trials. BMJ.

[29] Sterne JA, Hernán MA, Reeves BC, Savović J, Berkman ND, Viswanathan M (2016). ROBINS-I: a tool for assessing risk of bias in non-randomised studies of interventions. BMJ.

[30] National Institutes of Health (NIH). Study Quality Assessment Tools -- NHLBI, NIH. Before-After (Pre-Post) Studies with No Control Group. 2022. Available from: https://www.nhlbi.nih.gov/health-topics/study-quality-assessment-tools. Accessed December 2, 2022.

[31] Moola S, Munn Z, Tufanaru C, Aromataris E, Sears K, Sfetcu R, et al. Systematic reviews of etiology and risk. In: Aromataris E, Munn Z, eds. JBI Manual for Evidence Synthesis. Australia: JBI; 2020. 10.46658/jbimes-20-08

[32] Seifi K, Ebrahimi Moghaddam H (2018). The effectiveness of self-care program on the life quality of patients with multiple sclerosis in 2015. J Natl Med Assoc.

[33] Hartley S (2009). Developing a self-management and exercise model for people with multiple sclerosis. Int J Ther Rehabil.

[34] Gallien P, Rouxel A, Brunet I, Larget A, Deburghgraeve V, Nicolas B (2014). Fatigue et sclérose en plaques: expérience d’un séminaire d’éducation thérapeutique. Ann Phys Rehabil Med.

[35] Ehde DM, Elzea JL, Verrall AM, Gibbons LE, Smith AE, Amtmann D (2015). Efficacy of a telephone-delivered self-management intervention for persons with multiple sclerosis: a randomized controlled trial with a one-year follow-up. Arch Phys Med Rehabil.

[36] Bombardier CH, Cunniffe M, Wadhwani R, Gibbons LE, Blake KD, Kraft GH (2008). The efficacy of telephone counseling for health promotion in people with multiple sclerosis: a randomized controlled trial. Arch Phys Med Rehabil.

[37] Brittle N, Brown M, Mant J, McManus R, Riddoch J, Sackley C (2008). Short-term effects on mobility, activities of daily living and health-related quality of life of a conductive education programme for adults with multiple sclerosis, Parkinson’s disease and stroke. Clin Rehabil.

[38] Feicke J, Spörhase U, Köhler J, Busch C, Wirtz M (2014). A multicenter, prospective, quasi-experimental evaluation study of a patient education program to foster multiple sclerosis self-management competencies. Patient Educ Couns.

[39] Plow M, Finlayson M, Liu J, Motl RW, Bethoux F, Sattar A (2019). Randomized controlled trial of a telephone-delivered physical activity and fatigue self-management interventions in adults with multiple sclerosis. Arch Phys Med Rehabil.

[40] Thomas S, Thomas PW, Kersten P, Jones R, Green C, Nock A (2013). A pragmatic parallel arm multi-centre randomised controlled trial to assess the effectiveness and cost-effectiveness of a group-based fatigue management programme (FACETS) for people with multiple sclerosis. J Neurol Neurosurg Psychiatry.

[41] Oz HS, Oz F (2020). A psychoeducation program for stress management and psychosocial problems in multiple sclerosis. Niger J Clin Pract.

[42] Köpke S, Kern S, Ziemssen T, Berghoff M, Kleiter I, Marziniak M (2014). Evidence-based patient information programme in early multiple sclerosis: a randomised controlled trial. J Neurol Neurosurg Psychiatry.

[43] Köpke S, Kasper J, Mühlhauser I, Nübling M, Heesen C (2009). Patient education program to enhance decision autonomy in multiple sclerosis relapse management: a randomized-controlled trial. Mult Scler.

[44] Finlayson M, Preissner K, Cho C, Plow M (2011). Randomized trial of a teleconference-delivered fatigue management program for people with multiple sclerosis. Mult Scler.

[45] Calandri E, Graziano F, Borghi M, Bonino S (2017). Improving the quality of life and psychological well-being of recently diagnosed multiple sclerosis patients: preliminary evaluation of a group-based cognitive behavioral intervention. Disabil Rehabil.

[46] Graziano F, Calandri E, Borghi M, Bonino S (2014). The effects of a group-based cognitive behavioral therapy on people with multiple sclerosis: a randomized controlled trial. Clin Rehabil.

[47] Mathiowetz VG, Matuska KM, Finlayson ML, Luo P, Chen HY (2007). One-year follow-up to a randomized controlled trial of an energy conservation course for persons with multiple sclerosis. Int J Rehabil Res.

[48] Mulligan H, Wilkinson A, Barclay A, Whiting H, Heynike C, Snowdon J (2016). Evaluation of a fatigue self-management program for people with multiple sclerosis. Int J MS Care.

[49] Pöttgen J, Moss-Morris R, Wendebourg JM, Feddersen L, Lau S, Köpke S (2018). Randomised controlled trial of a self-guided online fatigue intervention in multiple sclerosis. J Neurol Neurosurg Psychiatry.

[50] Ghahari S, Leigh Packer T, Passmore AE (2010). Effectiveness of an online fatigue self-management programme for people with chronic neurological conditions: a randomized controlled trial. Clin Rehabil.

[51] Momenabadi V, Kaveh MH, Nakhaee N, Karimzadeh Shirazi K, Dastoorpoor M, Sedighi B (2019). Effect of educational intervention based on health-promoting self-care behaviors model on quality of life, resilience, and sense of coherence in patients with multiple sclerosis: a randomized controlled trial. Iran Red Crescent Med J.

[52] Miller DM, Moore SM, Fox RJ, Atreja A, Fu AZ, Lee JC (2011). Web-based self-management for patients with multiple sclerosis: a practical, randomized trial. Telemed J E Health.

[53] Albarqouni L, Glasziou P, Hoffmann T (2018). Completeness of the reporting of evidence-based practice educational interventions: a review. Med Educ.

[54] Rudd BN, Davis M, Beidas RS (2020). Integrating implementation science in clinical research to maximize public health impact: a call for the reporting and alignment of implementation strategy use with implementation outcomes in clinical research. Implement Sci.

[55] Albano MG, Jourdain P, De Andrade V, Domenke A, Desnos M, d’Ivernois JF (2014). Therapeutic patient education in heart failure: do studies provide sufficient information about the educational programme?. Arch Cardiovasc Dis.

[56] Cho MK, Kim MY (2021). Self-management nursing intervention for controlling glucose among diabetes: a systematic review and meta-analysis. Int J Environ Res Public Health.

[57] Khan F, Amatya B, Galea M (2014). Management of fatigue in persons with multiple sclerosis. Front Neurol.

[58] Tur C (2016). Fatigue management in multiple sclerosis. Curr Treat Options Neurol.

[59] Turner AP, Knowles LM (2020). Behavioral interventions in multiple sclerosis. Fed Pract.

[60] Hind D, Cotter J, Thake A, Bradburn M, Cooper C, Isaac C (2014). Cognitive behavioural therapy for the treatment of depression in people with multiple sclerosis: a systematic review and meta-analysis. BMC Psychiatry.

[61] Hart S, Fonareva I, Merluzzi N, Mohr DC (2005). Treatment for depression and its relationship to improvement in quality of life and psychological well-being in multiple sclerosis patients. Qual Life Res.

[62] Golay A, Lagger G, Chambouleyron M, Carrard I, Lasserre-Moutet A (2008). Therapeutic education of diabetic patients. Diabetes Metab Res Rev.

[63] Herek GM. Developing a theoretical framework and rationale for a research proposal. In: Pequegnat W, Stover E, Boyce CA, eds. How to Write a Successful Research Grant Application: A Guide for Social and Behavioral Scientists. Boston, MA: Springer; 2011. p. 137-45. 10.1007/978-1-4419-1454-5_12

[64] Al Slamah T, Nicholl BI, Alslail FY, Melville CA (2017). Self-management of type 2 diabetes in gulf cooperation council countries: a systematic review. PLoS One.

[65] Finlayson M, Holberg C (2007). Evaluation of a teleconference-delivered energy conservation education program for people with multiple sclerosis. Can J Occup Ther.

[66] Rasova K, Martinkova P, Pavlikoma M, Cattaneo D, Jonsdottir J, Henze T (2015). Physical therapy provision in multiple sclerosis across Europe: a regional lottery. Eur J Phys Rehabil Med.

[67] Marziniak M, Brichetto G, Feys P, Meyding-Lamadé U, Vernon K, Meuth SG (2018). The use of digital and remote communication technologies as a tool for multiple sclerosis management: narrative review. JMIR Rehabil Assist Technol.

[68] Buchanan RJ, Radin D, Huang C, Zhu L (2010). Caregiver perceptions associated with risk of nursing home admission for people with multiple sclerosis. Disabil Health J.

[69] Penwell-Waines L, Goodworth MC, Casillas RS, Rahn R, Stepleman L (2016). Perceptions of caregiver distress, health behaviors, and provider health-promoting communication and their relationship to stress management in MS caregivers. Health Commun.

[70] Petrikis P, Baldouma A, Katsanos AH, Konitsiotis S, Giannopoulos S (2019). Quality of life and emotional strain in caregivers of patients with multiple sclerosis. J Clin Neurol.

[71] Opara J, Jaracz K, Brola W (2012). Burden and quality of life in caregivers of persons with multiple sclerosis. Neurol Neurochir Pol.

[72] Shaikh MM, Nadar SK (2018). Peer-facilitated patient education: an underutilised resource. Sultan Qaboos Univ Med J.

[73] Bijani M, Niknam M, Karimi S, Naderi Z, Dehghan A (2022). The effect of peer education based on Pender’s health promotion model on quality of life, stress management and self-efficacy of patients with multiple sclerosis: a randomized controlled clinical trial. BMC Neurol.

