# Supplementary material for: A systematic review of the impact of therapeutic education programs on the quality of life of people with Multiple Sclerosis
Source: Health Promot Perspect. 2024 Jul 29;14(2):97–108. doi: 10.34172/hpp.42619 (PMC11403335; doi:10.34172/hpp.42619)
Supplement: Supplementary file 1 — contains the search strategy [file hpp-14-97-s001.pdf]

Raji et al, **Health Promotion Perspectives,** 2024, 14(2), 97-S1
doi: 10.34172/hpp.42619
https://hpp.tbzmed.ac.ir

**PUBMED**
(("Multiple Sclerosis"[Mesh]) AND ("Self-Management"[Mesh] OR "Patient Education as Topic"[Mesh] OR "Self Care"[Mesh])) AND "Quality of Life"[Mesh]
**SCOPUS**
TITLE-ABS-KEY (("Multiple Sclerosis" OR" MS" OR "Chronic Progressive Multiple Sclerosis" OR "Relapsing-Remitting chronic" OR "secondary progressive multiple sclerosis" OR "primary progressive multiple sclerosis" OR "relapsing remitting multiple sclerosis" OR "remitting-relapsing multiple sclerosis") AND ("therapeutic patient education" OR "patient education" OR "patient therapy" OR "therapeutic education" OR "education of the patient" OR "health education" OR "Counselling/methods" OR "patient information" OR "patient care planning" OR "self-management" OR "self care" OR "self-management program") AND ("HRQL" OR "QoL" OR "health-related quality of life" OR "Quality of Life" OR "QOL tools" OR "SF-36" OR "WHOQOL" OR "DQOL" OR "well-being" OR "psychological well-being" OR "emotional well-being" OR "SF-18" OR "SF-12")) AND (LIMIT-TO (PUBYEAR , 2022) OR LIMIT-TO (PUBYEAR , 2021) OR LIMIT-TO (PUBYEAR , 2020) OR LIMIT-TO (PUBYEAR , 2019) OR LIMIT-TO (PUBYEAR , 2018) OR LIMIT-TO (PUBYEAR , 2017) OR LIMIT-TO (PUBYEAR , 2016) OR LIMIT-TO (PUBYEAR , 2015) OR LIMIT-TO (PUBYEAR , 2014) OR LIMIT-TO (PUBYEAR , 2013) OR LIMIT-TO (PUBYEAR , 2012) OR LIMIT-TO (PUBYEAR , 2011) OR LIMIT-TO (PUBYEAR , 2010) OR LIMIT-TO (PUBYEAR , 2009) OR LIMIT-TO (PUBYEAR , 2008) OR LIMIT-TO (PUBYEAR , 2007))
**WEB OF SCIENCES**
(("Multiple Sclerosis" OR" MS" OR "Chronic Progressive Multiple Sclerosis" OR "Relapsing-Remitting chronic" OR "secondary progressive multiple sclerosis" OR "primary progressive multiple sclerosis" OR "relapsing remitting multiple sclerosis" OR "remitting-relapsing multiple sclerosis") AND ("therapeutic patient education" OR "patient education" OR "patient therapy" OR "therapeutic education" OR "education of the patient" OR "health education" OR "Counselling/methods" OR "patient information" OR "patient care planning" OR "self-management" OR "self care" OR "self-management program") AND ("HRQL" OR "QoL" OR "health-related quality of life" OR "Quality of Life" OR "QOL tools" OR "SF-36" OR "WHOQOL" OR "DQOL" OR "well-being" OR "psychological well-being" OR "emotional well-being" OR "SF-18" OR "SF-12"))

Supplementary file 1

Search strategy
