# Supplementary material for: A systematic review of the impact of therapeutic education programs on the quality of life of people with Multiple Sclerosis
Source: Health Promot Perspect. 2024 Jul 29;14(2):97–108. doi: 10.34172/hpp.42619 (PMC11403335; doi:10.34172/hpp.42619)
Supplement: Supplementary file 2 — Supplmentary file 2: contains the quality of studies [file hpp-14-97-s002.pdf]

Raji et al, **Health Promotion Perspectives,** 2024, 14(2), 97-S2
doi: 10.34172/hpp.42619
https://hpp.tbzmed.ac.ir

Supplementary file 2

Study	D1	D2	D3	D4	D5	Overall bias
OZ et al. (2020)	Low	Some concerns	Low	Low	Low	Some concerns
Kopke et al (2009)	Low	Low	Low	Low	Low	Low
Köpke et al. (2013)	Low	Low	High	Low	someconcerns	High
Miller et al. (2011)	Someconcerns	Some concerns	Low	Low	Low	Some concerns
Thomas et al. (2013)	Low	Some concerns	High	Low	Some concerns	High
Mathiowetz et al. (2007)	Low	Some concerns	Low	Low	Low	Some concerns
Momenabadi, et al (2020)	Low	Low	Low	Low	Low	Low
Plow, M et al (2019)	Low	Low	Low	Low	Low	Low
Finlayson et al (2011)	Low	Low	Low	Low	Low	Low
Ehde, et al (2015)	Low	Low	Low	Low	Low	Low
Graziano et al. (2014)	Low	Some concerns	Low	Low	Low	Some concerns
Ghahari, et al (2010)	Low	Some concerns	Low	Low	Low	Some concerns
Bombardier, et al (2008)	Low	Some concerns	Low	Low	Low	Some concerns
Pöttgen et all(2018)	Low	Low	Low	Low	Low	Low

Quality assessment

A. The Cochrane RoB 2.0 tool for Randomised controlled trials

D1 : Bias arising from the randomisation process
D2 : Bias due to deviations from intended interventions
D 3 : Bias due to missing outcome data
D4 : Bias in measurement of the outcome
D5: Bias in selection of the reported result

Judjement :
High
Some concerns
Low

B. ROBINS-I tool for for quasi-experimental studies

Study	D1	D2	D3	D4	D5	D6	D7	Overall bias
Feicke et al (2014)	Moderate	Moderate	Low	Low	Low	Low	Low	Moderate
Calandri,et al. (2017)	Low	Low	Low	Low	Low	Low	Low	Low

Domains
D1 :Bias due to confounding
D2: Bias in selection of participants into the study
D3: Bias in classification of interventions
D4: Bias due to deviations from intended interventions
D5: Bias due to missing data

D6: Bias in measurement of outcomes
D7: Bias in selection of the reported results

Judjement :
Low
Moderate
Serious

Critical

C. The National Institutes of Health (NIH) quality assessmenttool for before-after (Pre-Post) study with no control group

**Study**	**Q1**	**Q2**	**Q3**	**Q4**	**Q5**	**Q6**	**Q7**	**Q8**	**Q9**	**Q10**	**Q11**	**Q12**	**Total score**	**Décision**
Hartely et al (2009)	Yes	Yes	Cannot Determine	Yes	No	Yes	Yes	Not Applicable	Yes	Yes	NA	NA	60%	Fair Quality
Seifi et al (2018)	Yes	Yes	Yes	Yes	Yes	Yes	Yes	Yes	Yes	Yes	NA	NA	83%	Good Quality
Mulligan et al. (2016)	Yes	Yes	Cannot Determine	Yes	NO	Yes	Yes	Not Applicable	Yes	Yes	NA	NA	60%	Fair Quality

Abbreviations: Y, yes; N, no; NA, not applicable; NR, not reported,Q1: Was the study question or objective clearly stated?,Q2: Were eligibility/selection criteria for the study population prespecified and clearly described?,Q3: Were the participants in the study representative of those who would be eligible for the test/service/intervention in the general or clinical population of interest?, Q4: Were all eligible participants that met the prespecified entry criteria enrolled?, Q5: Were the participants in the study representative of those who would be eligible for the test/service/intervention in the general or clinical population of interest?; Q6: Was the test/service/intervention clearly described and delivered consistently across the study population?,Q7: Were the outcome measures prespecified, clearly defined, valid, reliable, and assessed consistently across all study participants?,Q8: Were the outcome measures prespecified, clearly defined, valid, reliable, and assessed consistently across all study participants?, Q9: Were the people assessing the outcomes blinded to the participants' exposures/interventions?, Q10: Was the loss to follow-up after baseline 20% or less? Were those lost to follow-up accounted for in the analysis?,Q11: Did the statistical methods examine changes in outcome measures from before to after the intervention? Were statistical tests done that provided p values for the pre-to-post changes?, Q12:Were outcome measures of interest taken multiple times before the intervention

D. The Joanna Briggs Institute (JBI) Critical Appraisal Checklist for analytical cross-sectional study

Study	Q1	Q2	Q3	Q4	Q5	Q6	Q7	Q8	Total score	Décision
Gallien et al (2020)	No	Yes	Yes	Unclear	Unclear	Unclear	Yes	Unclear	37%	Poor Quality
Brittle et all (2008)	Yes	No	Yes	Yes	Unclear	Unclear	Yes	Yes	62%	Moderate Quality

Abbreviations:NA: not applicable; U: unclear; Y: yes; Q1: Were the criteria for inclusion in the sample clearly defined?; Q2: Were the study subjects and the setting described in detail?; Q3: Was the exposure measured in a valid and reliable way?; Q4: Were objective, standard criteria used for measurement of the condition?; Q5: Were

confounding factors identified?; Q6: Were strategies to deal with confounding factors stated?; Q7: Were the outcomes measured in a valid and reliable way?; Q8: Was appropriate statistical analysis used?
